# Impact of green space on residents' wellbeing: A case study of the Grand Canal (Hangzhou section)

**DOI:** 10.3389/fpubh.2023.1146892

**Published:** 2023-03-30

**Authors:** Yuke Xia

**Affiliations:** The Grand Canal Cultural Institute, Gongshu District, Hangzhou, China

**Keywords:** green development, dynamic management, water quality, cultural heritage management, urban

## Abstract

**Introduction:**

In this study aimed to discuss the importance of the combination of cultural heritage management and green development for urban development by analyzing the upgrading and renovation of the Grand Canal (Hangzhou section) as a successful case. In recent years, green development has risen to prominence as a paradigm shift. Additionally, culture, as an engine to drive urban development, has received more attention.

**Methods:**

This research used a hybrid approach to examine the importance of combining green development with cultural heritage management. The qualitative method was an interview analysis of 13 residents living in the Hangzhou section of the Grand Canal. Based on the analysis of multiple water quality variables in Hangzhou from 1998 and 2014 to 2021, the empirical results proved that it is feasible to integrate green development (environment and economy) into the cultural heritage management of the case study area.

**Results and discussion:**

The results further prove that only through an understanding of the relationship between cultural heritage and green development can a virtuous cycle of development be created, thereby promoting the continuous development of a unique and historically significant urban area. The results of this study suggest that, in the development of mega-cities, although the preservation and inheritance of historical and cultural heritage conflicts with the green development of modern cities, a successful example has been explored in Hangzhou, including grassroots governance efforts like Gongshu District. There, the two factors can be mutually compatible and promote each other, enhancing the well-being and happiness of local residents.

## 1. Introduction

The significance of green development is clear from the considerable research exploring its determinants, sustainability (1), Green New Deal (2), and ecological civilization construction (3). Despite these studies discussing the influencing factors of a green economy, more attention should be paid to the connection between culture and green development. After the Rio Summit (4), the global society has been facing a dual challenge: expanding economic opportunities and addressing environmental pressures. Green development is the intersection of these two challenges, achieving a combination of both. Green development is a paradigm innovation based on traditional development (5), which takes environmental resources as an intrinsic element of economic development (6). Green development is intended to ensure sustainable economic, social, and environmental development. It continues to provide the environmental resources and services upon which the wellbeing of the public depends.

There is a close connection between green development and cultural resources. In the practice of green development, the protection and utilization of cultural resources are two very important aspects.^1^ According to the Free Dictionary, green development refers to an approach to sustainable development^2^ that considers environmental concerns and the efficient use of resources, such as land, energy, and water, and also involves the preservation of cultural and archeological resources. By incorporating environmental responsiveness, green development promotes the wellbeing of the environment and the surrounding communities. This approach to development not only benefits human and natural communities but also promotes cultural development. Additionally, green development is economically viable, providing a sustainable and responsible path for development (7). While remaining economically viable, green development benefits human and natural communities as well as cultural development. By integrating the cultural heritage with green development programs, such as environmental protection measures, national legislation outlines steps to preserve cultural heritage. Furthermore, for culture, green represents sustainability, i.e., the sustainability of cultural development is related to the preservation of cultural beliefs, cultural practices, cultural heritage preservation, culture as its entity, and any particular culture in the future (8–10).

Several studies (9, 10) on cultural heritage management have been published, examining the links between continued exposure to surrounding green development and cultural heritage management. Several studies found an inevitable need in the current socio-economic environment, incorporating the concept of green development in urban development, integrating with the city's cultural heritage, and reinvigorating the links between the urban cultural heritage and its natural environment (11). Most studies investigated the connection between the environment and cultural heritage, tracking how to perceive the role and importance of culture in green development. National and international efforts to promote green development as a new approach to manage cultural heritage have been intensifying.

It is important to consider a range of perspectives and factors when examining the links between the surrounding green development and cultural heritage management. This can help ensure that the decision-making processes are inclusive, equitable, and considerate of a broad range of values and priorities. The links between the surrounding green development and cultural heritage management are complex and multifaceted, and there are several aspects that are often overlooked or left out of the discussion. The preservation of cultural heritage and green spaces is often linked to broader historical and systemic factors. Examining the links between these factors and the management of green development and cultural heritage can help identify and address the underlying root causes of issues.

In December 2015, the International Conference on “Culture for Sustainable Cities” organized by the United Nations was held in Hangzhou, using the “Hangzhou Outcomes” to focus the spotlight on the power of culture for sustainable urban development (11). Its effects make the management of cultural heritage more challenging. The successful construction of Hangzhou can be attributed to its focus on sustainable development, cultural preservation, and internationalization, all of which have contributed to the city's economic growth and high quality of life for its residents; thus, the construction of Hangzhou is considered to be a successful model of coastal open cities in China. The Grand Canal is the greatest masterpiece of hydraulic engineering achievement in the history of society, creating the largest project in the world (12). In 2014, the Grand Canal was successfully inscribed on the World Heritage List (12).

The Grand Canal was built gradually over a long period, and its introduction is complex and not simple. It is a huge inland waterway system, running from Beijing in the north to Zhejiang province, Hangzhou city in the south, crossing eight provinces (12). It has contributed significantly to China's economic stability and prosperity over the eras and is still one of the main internal communication channels at present (12). Chinese President Xi Jinping has emphasized that “The Grand Canal is a precious heritage left to human beings by our ancestors, and it is a flowing culture, which should be well protected, inherited, and utilized” (13). Xi also emphasized that the preservation of the Grand Canal's cultural heritage should be integrated with the promotion of ecological and environmental protection, the safeguarding and restoration of renowned cities and towns along its route, the development of cultural tourism, and the modernization of canal navigation. These efforts aimed to create favorable conditions for the economic and social progress of the regions along the Grand Canal and to enhance the wellbeing of the people who live there (13).

The Grand Canal (Hangzhou section) has been chosen as a case study for this research since such a comprehensive conservation initiative has successfully integrated cultural heritage management and green development into sustainable urban development and has come to serve as an excellent model. In fact, the Grand Canal's other sections have accomplished well in terms of conservation. This research concentrates on analyzing the Hangzhou section due to space limitations and the need for the author's observations.

Despite the cultural projects' potential to stimulate local economic development, less attention has been given to integrating the existing cultural heritage management with green development. This research is structured into three parts. First, it provides a review of the current theory of green development. Second, by analyzing the policy background of Hangzhou and the discourse of the Grand Canal construction (Hangzhou section), this research clarifies how local government engages in green development. The third part sheds light on how the local government made relevant policies to integrate green development into the cultural heritage management of the case study area and the unique kind of green development represented by these specific cultural heritage sites will be examined. This research will also include interviews with Grand Canal residents and the use of empirical cases to demonstrate the importance of integrating green development with cultural heritage management. The authors argue that this case study is highly replicable and can serve as an exemplar for other countries seeking to integrate cultural management of the Grand Canal heritage with urban green development.

## 2. Literature review

### 2.1. Green development

“Green development” generally refers to a sustainable approach to urban planning and development that aims to minimize environmental impact and promote ecological sustainability (14). The 1960s and 1970s saw a significant increase in awareness and concern about environmental issues, particularly with the publication of Rachel Carson's book “Silent Spring” in 1962 (15). This development helped to catalyze the modern environmental movement and led to the first Earth Day in 1970, which mobilized millions of people to call for greater environmental protection (16). Western societies first began to pay attention to the relationship between economic growth and environmental capacity in the late 17^th^ century, and this period was the germ of the idea of green development (17). A new stage in rebuilding political discourse in response to ecological problems and environmental movements is represented by green growth and a green economy (18). As noted by the emergence of “ecological economics” (19) and moral condemnation and criticism of the industrial civilization, such as *A Blueprint for Survival* (20), those decades saw the mainstreaming of environmentalism. Green development can include measures such as using renewable energy sources, improving energy efficiency, reducing waste and pollution, preserving green spaces, and promoting public transportation and alternative modes of transportation that minimize carbon emissions (21).

In 1972, the United Nations conference was held in Stockholm, the first world conference to address the environment as a major issue (22). The Brundtland Commission, supported by the UN, defined sustainability as fulfilling current requirements without jeopardizing the needs of future generations in terms of development, marking the official beginning of the concept (23). At the Rio Earth Summit, it took center stage as the mainstream environmental movement's ethos and slogan (4). Its critics dismissed it as a trendy phrase to which everyone refers to; yet, nobody can define it (24). Some have questioned the promise of a new paradigm as mere green propaganda for business as usual (25). Others viewed it as a hegemonic “cover-up operation” designed to persuade and calm a populace concerned about the impact of economic growth (26). It is most peculiar that ecological concepts have been overridden so that, by the 2000s, sustainable development focuses almost exclusively on economic growth (25). The rise in environmental concerns is a significant factor that led to the emergence of green development (27). Sustainable development failed to address the growing environmental issues and the urgent need to mitigate the effects of climate change (28). Therefore, green development emerged as a new framework that prioritizes environmental protection and addresses the shortcomings of sustainable development (29). Despite the efforts of sustainable development, the world continues to face pressing environmental challenges such as pollution, loss of biodiversity, and climate change. Sustainable development discourse and projects have not been successful in achieving their goals. Green development has emerged as an alternative approach that provides a fresh perspective and solutions to the current environmental issues (17). Green development represents new approaches to environmental intervention, such as “a particular type of capital which must be measured, conserved, produced, and even accumulated” (30). The Global Green Growth Institute identifies green development as one of the key pillars of sustainable and inclusive economic development, defining it as a development way aimed at achieving eco-friendly and economic growth (31).

### 2.2. Green development under the Chinese context

Green development has become an important concept in China's development strategy (32). In recent years, China has taken significant steps toward reducing pollution, increasing the use of renewable energy, and promoting sustainable development (33).

The concept of green development was formally proposed at the Fifth Plenary Session of the 18^th^ CPC Central Committee (34). In recent years, the green development research has received increasing attention and recognition. Scholars generally agree that the green development model has become the inevitable choice for China's future development, focusing on ecological conservation and transforming the industry through high-quality green development (35). According to some studies, China's green development is a more innovative and upgraded development model to achieve sustainable development by protecting the ecological environment under the constraints of ecological and environmental capacity (36). Green development places a greater emphasis on ecological priorities and requires more systematic, holistic, and coordinated linkages between economic, social, and natural systems (37). Green development is a sustainable approach to development that prioritizes ecological considerations (38). It recognizes that economic growth and social progress must be achieved in harmony with nature and requires a more systemic, holistic, and coordinated approach to achieve this balance. This means that green development aims to ensure that economic activities are carried out in ways that are environment friendly and do not degrade natural resources. Green development recognizes that human society is an integral part of nature and seeks to promote social progress and economic growth while preserving the integrity of natural systems (39). This requires a more systematic approach to planning and policymaking, which takes into account the interrelationships between economic, social, and natural systems. Therefore, it is necessary to examine green development based on more complex spatial and temporal conditions and specific contexts (40). China's green development goals are backed by a strict environmental conservation law (41) and the government has launched a series of action plans to combat air, water, and soil pollution (42).

### 2.3. Cultural heritage management

Studies of cultural heritage are receiving increasing international attention as a result of the longstanding efforts of the United Nations Organization (43). In transforming our world Agenda for sustainable development, reference is made to culture as a priority component of urban planning and strategies. It is worth noting that this is also mentioned in the New Urban Agenda (44). “Tradition” has become a rare resource of time, valued, and turned into an “inheritance” (45). It seems to have evolved through a process generally sparked by the rediscovery of cultural values (46). The 17th UNESCO Conference in Paris focused on the serious problem of the destruction of cultural and natural heritage, developed a series of measures for its protection, interpreted cultural and natural heritage, and defined cultural objects, architectural complexes, and sites as cultural heritage (47).

In particular, in defining topographical areas as human works of outstanding universal value or type of nature and humanity in historical, esthetic, ethnographic, or anthropological terms (47). The International Committee on Archaeological Heritage Management outlines fundamental guidelines for the examination, upkeep, and conservation, as well as the reconstruction of architectural heritage based on inventories and broad assessments of the resources (48). It was determined that this Committee should include the correct long-term conservation and curation of all relevant records and collections and the preservation of monuments and places *in situ*. The idea of preserving the heritage in its native setting is violated by any transfer of heritage components to new locations (49). Ensuring cultural content and diversity, as emphasized in the Recommendation on the Safeguarding of UNESCO (50) and United Nations (51) issued by the UNESCO (52), the United Nations adopted the text of the Convention for the Safeguarding of Intangible Cultural Heritage (53). Two of those points for managing intangible cultural heritage are mentioned: (i) intangible Cultural Heritage Preservation and (ii) ensuring that intangible heritage is treated with respect by the communities, groups, and individuals involved.

The dynamic management that will be mentioned in the cultural heritage management process is closely related and both involve the management of resources in a dynamic and ever-changing environment (54). Dynamic management is the process of actively and continuously adapting and adjusting management strategies and practices in response to changes in the environment, market conditions, or other external factors. In the context of cultural heritage management, dynamic management involves adapting strategies and practices to ensure the preservation, protection, and promotion of cultural heritage resources under changing social, economic, and environmental conditions (55). Dynamic management is a crucial aspect of cultural heritage protection because it enables cultural heritage sites to adapt and evolve over time while preserving their significance and authenticity (56). Dynamic management is a crucial aspect of cultural heritage protection (57). By embracing this approach, cultural heritage sites can continue to evolve and adapt while preserving their significance and authenticity for future generations (57).

### 2.4. The relationship between the green development and cultural heritage

Green development and cultural heritage are two interrelated concepts that can have a significant impact on each other. Green development refers to the approach to economic growth that emphasizes sustainability and the use of renewable resources to meet the needs of the present generation without compromising on the ability of future generations to meet their own needs. Cultural heritage, on the other hand, refers to the collective identity of a community or society, which includes traditions, beliefs, customs, and artifacts that have been passed down from one generation to another. The relationship between the green development and cultural heritage can be understood in several ways.

Intangible cultural heritage has the quality of cultural diversity and is also an essential guarantee for sustainable development; cultural heritage management has become a universal aspiration and a common concern (53). The cultural heritage reflected in the World Heritage List is regularly localized in cities (58). Continuity and compatibility are the greatest demanding situations for heritage management, as the historic environments need to constantly break barriers in form and function (59). Although it is rare, a multidisciplinary study on the connection between cultural heritage management and green development has caught the interest of specialists (60–62). The preservation of cultural heritage can contribute to green development by promoting the use of sustainable practices and the protection of the environment. The cultural heritage has been dexterously preserved over the historical (63) phase of unprecedented and continuous green development (64), the importance of promoting knowledge about green development, and the potential for results by fusing the use of cultural heritage with its inherent values, which has been highlighted by the UNESCO World Heritage Center and related organizations (65, 66).

The most remarkable feature of the World Heritage Convention (67) is that it links the concepts of the conservation of nature and the conservation of cultural property and presents them in a single list. The Convention recognizes how humans interact with nature and the essential need to maintain a balance between the two. The Budapest Declaration (68) adopted stresses the need to ensure an appropriate and fair balance between conservation, sustainability, and development, so that world heritage can be protected to promote social and economic development and the quality of life in our communities (68). In addition, in 2005, the Operational Guidelines further recognized that world heritage sites could support ecologically and culturally sustainable uses (69).

**Figure 1:**
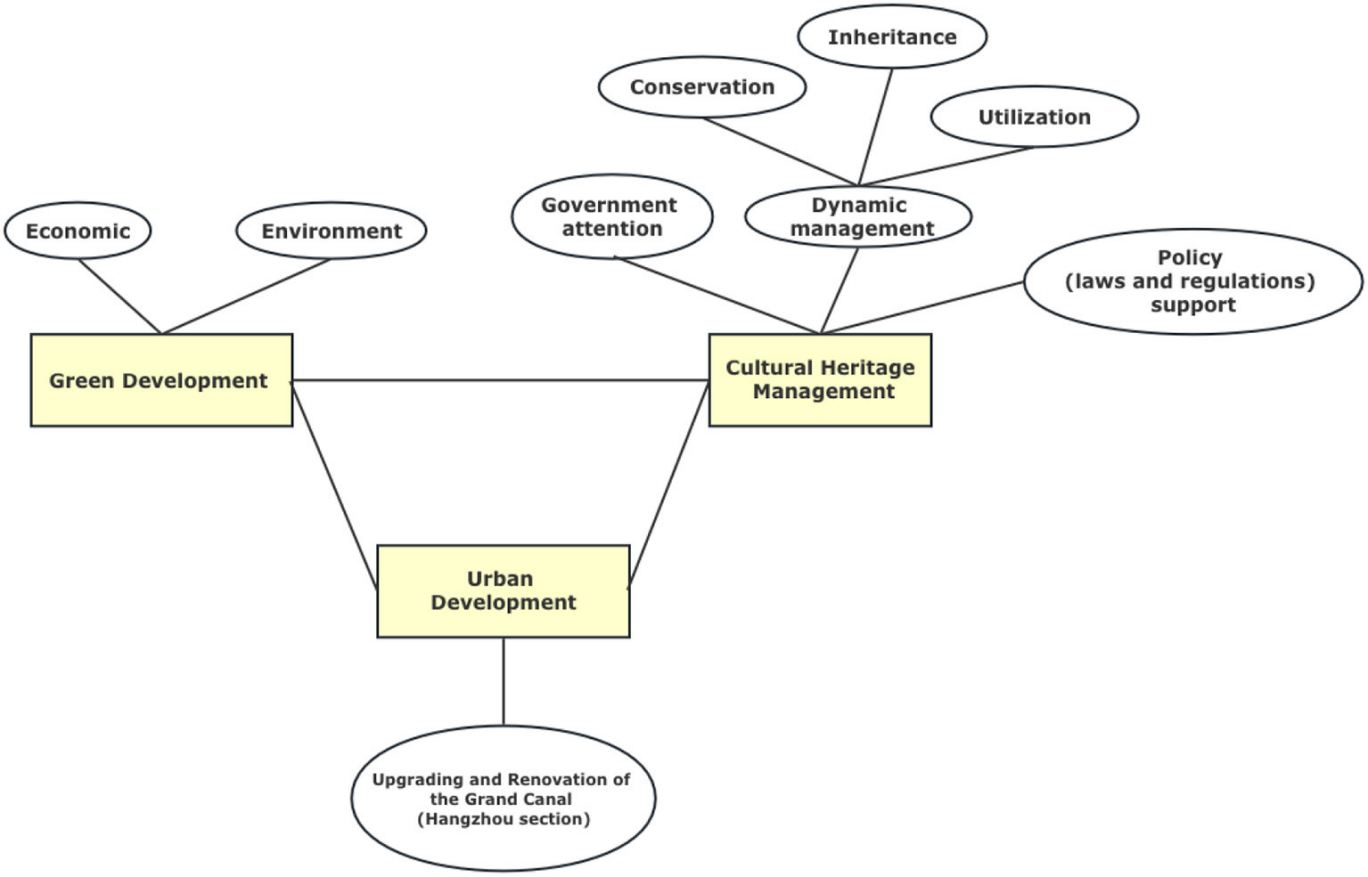
Research model.

Culture and cities are so closely linked that the 2030 Green Development Agenda incorporates culture (70). The Union of Cities and Local Government (UCLG) has also adopted the idea that culture has an irreplaceable role in urban development, aiming to fill the gap between cultural management and green development (71, 72). An integrated and shared global framework is still missing to provide practical and standardized guidelines to address the integration of these topics (73, 74). The Research model of this study is shown in Figure 1.

## 3. Methodology

### 3.1. Case study

Admittedly, the use of case studies in research implies that comprehensive and integrated problems can be addressed, and that research approach allowed the researcher to narrow down a complex and broad subject matter or phenomenon into manageable research issues (75). The researcher obtained a more in-depth analysis of the phenomenon by collecting both qualitative and quantitative data sets about it (76). In a case study, the researcher collected a variety of data, including quantitative and qualitative information, from multiple sources such as interviews, observations, documents, and other relevant materials. The goal was to gain a comprehensive understanding of the case being studied and to identify patterns, themes, and insights that can help to explain and interpret the findings.

#### 3.1.1. Cultural heritage of the Grand Canal (Hangzhou section)

Flowing for thousands of years, the Grand Canal is not only a “treasure of the world” but also the “lifeblood of the city” of Hangzhou (77). Since 2002, canal improvement and conservation have been listed as one of the top 10 construction projects in Hangzhou (78). The “Hangzhou Grand Canal Cultural Protection, Inheritance and Utilization and National Cultural Park Construction Plan” put forward specific requirements: focusing on the construction of four types of main functions: control and conservation areas, theme exhibition areas, cultural and tourism integration areas, and traditional utilization areas (79).

The Grand Canal (Hangzhou section) has 11 heritage site segments mainly concentrated in Gongshu District (78). Among them, six heritage sites are as follows: Gongchen Bridge (80), Guangji Bridge (81), Qiaoxi Historical District (82), Fengshan Water Gate Site (83), Fuyi Granary (84, 85), and Xixing Canal (86). Five sections of heritage rivers are (87) Hangzhou Pond (south section), Shangtang River (Hangzhou section), Middle River, Longshan River, and Hangzhou Section of Zhendong Canal (88) (see Table 1).

**Table 1:** Some heritage elements of the Grand Canal (Hangzhou section).

**Heritage elements**		**Basic information**
**Categories**	**Name**	
River	Shangtang River (上塘河)	Formerly one of the sections of the Lingshui Road (陵水道) built around 210 B.C., it was the main channel at the southern end of the Grand Canal until the 14^th^ century A.D. after the full length of the Grand Canal was opened in the 7^th^ century A.D.
	Middle River (中河)	It was built in the Tang Dynasty and runs north-south through the middle of Hangzhou city, connecting Shangtang River (上塘河) in the north and Longshan River (龙山河) in the south and is now a city landscape road.
	Longshan River (龙山河)	It was first dug in the 10^th^ century AD, connecting the Grand Canal with the Qiantang River and completing the Qiantang River transport port.
	Xixing Canal (西兴运河)	It was first dug in 307 AD. It became an important section of the East Zhejiang Canal.
Remains of hydraulic facilities	Guangji Bridge (广济桥)	Built in 1489 AD, the bridge is 78.7 meters long and is well preserved.
	Gongchen Bridge (拱宸桥)	Built in 1631 A.D., it is 98 m long and is well preserved.
Accessory remains	Fuyi Granary (富义仓)	It is an ancient urban public storage complex along the Grand Canal that is relatively well preserved. It was first built in 1880–1884 AD. Now the basic pattern still exists.
Related Heritage	Qiaoxi Historical District (桥西历史文化街区)	Located in Gongshu District, it is an urban residential area formed by relying on the geographical advantage of Gongchen Bridge as a major water and land transportation route. Now the pattern of the historical district is well preserved.

#### 3.1.2. Policy support

The Zhejiang Provincial Government has proposed the “Five water cohabitation” concept (五水共治), which aims to improve Zhejiang Province's water environment comprehensively while promoting people's wellbeing (89). It is necessary to explain that “sewage treatment,” “flood prevention,” “drainage water,” “water supply treatment,” and “water conservation” (90) are all maintained by the Five Water Cohabitation, and Hangzhou has since implemented a number of policies to improve its aquatic environment. The “Planning Outline for Grand Canal Cultural Protection, Inheritance and Utilization,” released in 2019 (91), clarified that it is necessary to build the main axis to drive the overall development and reshape the Grand Canal in accordance with the idea of protecting the cultural heritage of the canal, improving the water resources of the canal, building a green ecological corridor, and promoting cultural tourism (92). The government set up the necessary departments to thoroughly manage the Grand Canal Heritage and its tributaries, restore the river's shoreline and the surrounding environment, and create an ecologically sound continuous riverfront space (93). In 2020, the construction of the Grand Canal national parks was included in the 14^th^ Five-Year Plan (94). Since then, it has moved into a new era of Grand Canal protection and inheritance. Based on the principle of culture-centeredness and priority of conservation, the plan set goals such as optimizing the system's structure, improving the local ecosystem, demonstrating cultural values, and strengthening the protection, demonstration, and utilization of the Grand Canal heritage (94). In 2021, “The Great Wall, the Grand Canal, and the Long March National Cultural Park construction and protection plans” (94), put forward specific requirements to strictly protect and manage all kinds of cultural relics and the surrounding environment, and protect the ecology and traditional culture (95).

### 3.2. Data collection

This research collected the Grand Canal (Hangzhou section) in China as research samples, and the data were obtained from trustworthy channels to ensure the credibility of the research. Given the integrity and continuity of panel data, the samples with serious missing data were not included. The data were primarily based on the Hangzhou Environmental and Ecological Bulletin. Statistical bulletins from 2014 to 2021 were referenced.

This research gathered data through regular work, including surveys and information on the preservation and renovation of the Grand Canal from local authorities, the media, and academic researchers, and also spent significant time correlating and analyzing these findings to draw more objective and credible conclusions to support the research arguments.

### 3.3. In-depth interview

The in-depth interview method is a qualitative research method. The researcher conducted personal and in-depth interviews with informants. During the interviews, research information was collected that cannot be captured in quantitative studies (96). In the process of interviewing, a similar interview outline was often used for a small group of individuals in a specific category, and then, the interviewer's pre-determined questions were revealed by summarizing the content of different interviewees' responses (97). Yet, there were many drawbacks to this method, such as the representativeness of the interviewees, the relevance of the interview outline, and whether the interviewees' personality characteristics influence the interviewer's judgment (96).

First, the researcher discovered 13 Informats who were qualified for the interview (or collecting public information from nearby residents interviewed). The interview information used in this study was also taken from public interview transcripts of studies of a similar type carried out by other media. These interviews were conducted with residents of the Grand Canal of various ages and genders to learn more about how their quality of life has changed over time, how satisfied and happy they feel in their community, and other related topics. The interview version also contrasted the alterations that took place before and after the Grand Canal renovation.

### 3.4. Research ethics

In this research, certain Informats may face potential risks, such as privacy and security issues. Consequently, Informats were anonymized in this study, and their real-life and online identities were guaranteed privacy. Informats were referred to as “Informat 1,” “Informat 2,” etc., during the data gathering process. All study Informats are anonymous. During the interviews, and to ensure reliability and critical distance, the researcher needs to maintain a neutral stance and not elicit answers from Informats.

## 4. Finding and discussion

**Figure 2:**
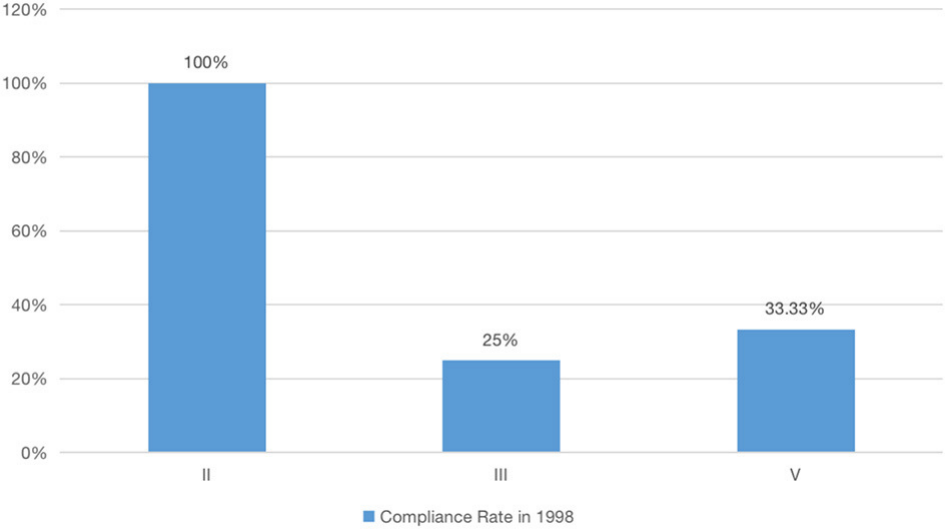
Water environment standard compliance rate in 1998.

The upgrading of the Grand Canal (Hangzhou section), a project that integrates green development and cultural heritage management, is discussed. The first one is about the emergence of green development. In this research, through the analysis of the data published in the Bulletin of Environment of Hangzhou, although the rate of attaining the standard of the primary protected area of surface water sources for domestic drinking water was 100% in 1998, the rate of attaining the standard of the secondary protected area of surface water sources for domestic drinking water was only 25%, and the rate of attaining the standard of the agricultural water area and the general landscape requirement waters was 33.33% (see Figure 2). To eradicate the water pollution of the canal, Hangzhou has been carrying out large-scale interception and treatment of sewage and canal comprehensive improvement since 1998. However, there are still many sources of pollution along the canal that are directly discharged into the canal, seriously endangering the quality of the canal water bodies. Hence, residents living close to the Grand Canal (Hangzhou section) used to struggle with the poor quality of water.

Level II water quality by conventional purification treatment (such as flocculation, sedimentation, filtration, and disinfection) can be used for life after drinking.

Level III water quality after treatment can also be used for life after drinking.

Below level III water quality is poor, and it cannot be used as a source of drinking water.

“*In the 80s, I often took a boat with my parents from Wulinmen and walked along the canal, the water was black and had a strong stench. Once the canal water, the most intuitive feeling is that the water is turbid and smelly.”*- Informat 3

“*Various factories along the canal discharged indiscriminately, “colorful” sewage is integrated into the Grand Canal, so the Grand Canal has become a stinky river that everyone dislikes. Before 2000 due to pollution, the water is basically extinct fish and shrimp, not to mention fishing, and even people can not get close to the front. The river stinks in the summer.”*- Informat 5

The Grand Canal's water quality was indeed severely polluted before the year 2000, according to the confirmation from Informats who live nearby.

“*In my childhood memories, the banks of the Grand Canal “scattered and dirty”, sewage can be discharged directly into the canal, the river is dyed black. At that time, the canal and the ditch is no different, even in winter, also emits a pungent smell.”*- Informat 12

“*Black pollutants floated on the water of the canal, and since the 1950s, industrial and storage businesses have crowded its banks. In the 1980s, this also factories on both sides of the canal brought more industrial and domestic sewage to the hundreds of tributaries that feed into the canal.”*- Informat 4

This is corroborated by the remarks of interviewees 12 and 4. In the wave of economic development, the water quality of the Grand Canal has become more polluted, with a series of water pollution incidents and a deteriorating water environment, which has affected the production and life of local residents to varying degrees.

**Figure 3:**
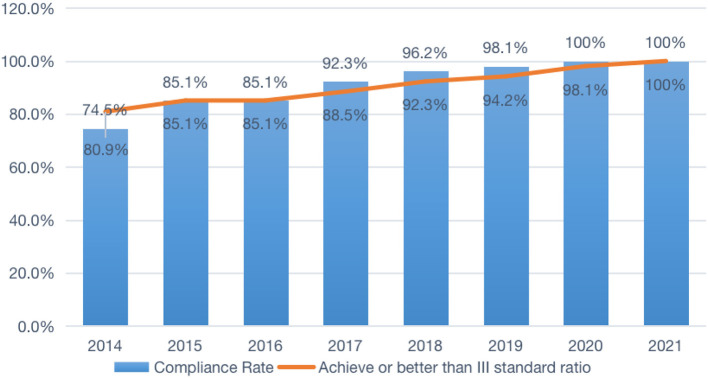
The water environment-related indicators of the Hangzhou city between 2014 and 2021.

One of the main goals of the renovation project is to improve the canal's water quality. Over the years, the canal has suffered from pollution caused by industrial and agricultural activities in the region. The Hangzhou government has implemented a number of measures to combat this pollution, such as limiting the discharge of wastewater into the canal and planting vegetation along its banks to help filter out pollutants. By comparing the water quality data of Hangzhou in the past 8 years, it was found that Hangzhou's water quality compliance rate continues to improve every year. The compliance rate increased by 19.1% from 2014 to 2021, and achieving or better than the III standard has been maintained at 100% for the past two years, which is 25.5% higher than the 74.5% in 2014 (refer to Figure 3). Positive environmental impact benefits everybody by reducing pollution and protecting aquatic ecology.

“*In recent years, it is obvious that the ecological quality around the canal has improved, and the nearby greenery feels all the greener, and it feels like the quality of life has improved.”*- Informat 9

People's wellbeing and green development are intricately linked as the health of the environment and the wellbeing of individuals are interdependent. Green development can help to protect the environment and ensure that natural resources are conserved for future generations. This is particularly important in the face of climate change, which poses a significant threat to both the environment and human wellbeing. By prioritizing sustainable practices and reducing our impact on the environment, we can create a healthier, more equitable, and sustainable future for all.

The local government further promoted the comprehensive protection projects of the Grand Canal. For example, the renovation of Old Residential Areas, old industrial plant reformation, and the renovation of urban-village, demolishing the illegal building (80), “Five-water Symbiosis” strategic project, “Sewage treatment,” the coordination of the relocation of highly polluting enterprises away from the banks of the Grand Canal, and the strict prohibition of urban construction and industrial development on the large-scale transformation of the natural landscape of the Grand Canal.

**Figure 4:**
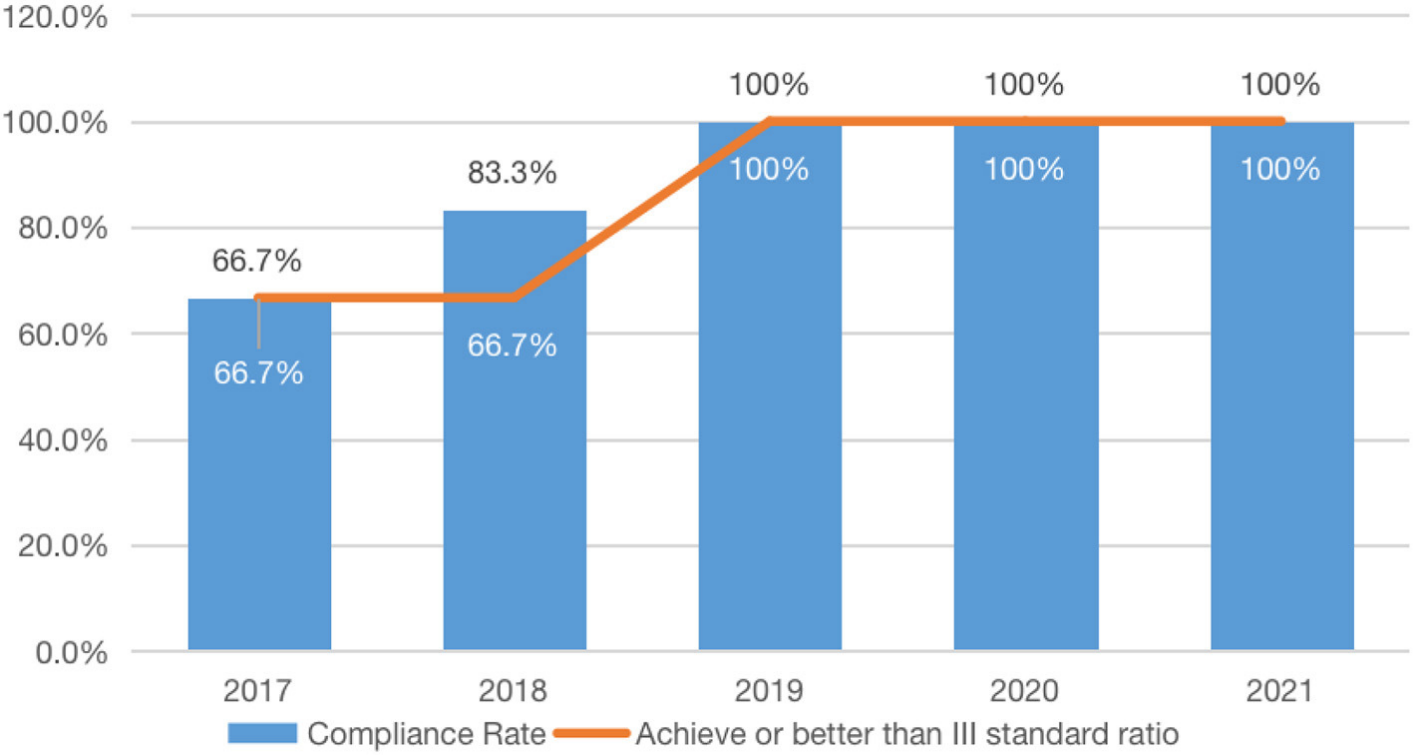
The water environment-related indicators of the Grand Canal (Hangzhou section) between 2017 and 2021.

The rate of meeting the criteria continues to increase each year, according to a comparison of Hangzhou Canal's water quality data during the past 5 years. The chart shows that, from 2017 to 2021, achieving or better than the III standard has been maintained at 100%, an increase of 33.3% from 2017 to 2021. Especially in the past 3 years, the water quality condition of the canal has continued to be excellently maintained at 100%, which has greatly changed the living space, production space, and ecological environment of the people, and their sense of wellbeing has been continuously improving (refer to Figure 4).

“*The clear water of the canal flowed from Gongchen Bridge, and rows of trees fell in golden color on the canal embankment road. The forest is full of color in autumn, and in summer, the flowers bloom not far from Xiaohe Park. I was able to catch such beautiful scenery.”*- Informat 12

The second is the protection, inheritance, and utilization of the Grand Canal (Hangzhou section) combined with cultural heritage and green development. For example, since the end of the last century, Hangzhou has entered a phase of rapid development, and many old factory buildings that have lost their productive functions and economic benefits have occupied numerous land resources. The core of the entire protection and improvement activity along the canal was centered on the remains of historic industry structures. These old industries, which are closely linked to the Grand Canal and have experienced the contemporary industrial growth of Hangzhou and carried the memories of a generation, are an essential component of the Grand Canal's cultural history and must be protected. In response to this urban issue, the government proposed to “give the river back to the people” to enhance and protect development around the canal. Additionally, green corridor components should be added as essential urban walking and natural infrastructure. These plots of old factory buildings should also be used and given cultural functions adapted to contemporary urban life.

“*I wander along the banks of the Grand Canal, and now I can no longer hear the roar of factory machinery or see the busy workers, but see the museum with an intense cultural atmosphere, which injects vitality and vigor into the old factory buildings. It feels like the old factory has completed its magnificent transformation and become a new cultural card along the canal.”*- Informat 10

Government efforts are also being made to preserve the cultural and historical significance of the canal. The Hangzhou government has invested in the restoration of historical sites along the canal, such as temples and bridges, as well as in improving the infrastructure and facilities for visitors. The renovation project also includes the construction of new pedestrian and cycling paths, which will provide a more accessible and environment-friendly way for people to explore the canal and its surrounding areas.

“*Trading the scenery with moving steps”*. This is the feeling raised by Informat 6. “*Living around the Gongshu section of the Grand Canal, she walks along the river every day after dinner.” 21 museums, 5 historical districts, small river parks, riverside green space...living here makes me feel blessed*.

The relationship between the old factories and the canals needs to be reshaped to activate this industrial heritage. In the past, fences separated the old factory buildings from the canals. The first step in the transformation was to remove the walls and break the boundaries. Canal industrial heritage has its unique image and spatial characteristics. It involved the preservation of chimneys, tall windows, and other traditional manufacturing building features; retaining the remaining tanks, pipes, hangers, and other structures in the site, and integration of original contemporary landscape design into the new architectural and site context.

“*I reside close to Xiaohe Park, which was formerly an oil depot as you can see. The oil depot pollutes the land and surrounding water sources. But since the oil depot was completely shut down in 2019, it has been transformed into Xiaohe Park with a beautiful environment. The quality of life has greatly improved.”*- Informat 11

“*The one I can think of the most is the Hangzhou Iron & Steel Factory, which was shut down after over 60 years of operation and has since been converted into a section of the Grand Canal National Cultural Park. It has retained numerous significant remains with great industrial features. This news made me incredibly delighted from the bottom of my heart.”*- Informat 13

Third, Hay (98) defines the establishment of green corridors as “green landscape chains connecting open places.” These canals with natural elements integrated ecology, culture, and recreation. Ahern (99) defines corridors as planned, designed, and managed linear network systems with ecological, cultural, recreational, and esthetic functions and as a sustainable land planning tool. The concept of heritage corridors was first introduced to China by Zhifang and Peng (100) a new approach to regional preservation of linear cultural heritage, often with a specific economic center, thriving tourism, modifying old buildings, environmental improvements, and entertainment. It is a new type of green corridor that integrates ecological, economic, historical, and cultural functions developed from green corridors that focus on ecological functions combined with heritage conservation. For example, the green corridor construction of the canal is more multifunctional through walking trails and public spaces all through the line, adding comprehensive service facilities.

“*Cycling along the canal a few years ago, it was evident that the greenway system could have been better. Now, the cycling path has become smooth. The greenery on both sides of the canal is doing well; I still remember that in autumn, cycling along the canal, the fragrance of osmanthus drifted.”*- Informat 7

When asked these questions, whether it is Xiaohe Park or it is the Hangzhou Iron & Steel Factory, these sites can be considered as epitomizing the cultural heritage management and green development of the Grand Canal. Although the sample is small, it gives a side view of the residents' future direction for canal city, and one of their expectations is the integration of green and culture.

“*I don't know about other places, but take Hangzhou as an example. At the beginning of the reform and opening up, the canal along the convenient transportation, many industries are also located nearby, the economy is relatively developed, and foreign exchanges are also convenient. But the industrial development of that time is serious environmental pollution. But now, the economy has been transformed, relying on the resources of the Grand Canal, and many cultural and creative-related or innovative stores have been opened here. And also often co-organize meaningful activities with the museum”* – Informat 11

“*The canal city has formed ecological landscape belts and cultural tourism resource belts on both sides, and emerging industries such as cultural creativity and technological design have sprung up. You can see a lot of unique coffee houses, bookstores, and tea bars opened along the canal. Many people will come here on weekends. And the surrounding area relies on the industrial heritage to carry out many special activities, such as various exhibitions. These activities add a youthful vitality to the old canal.”*- Informat 1

The construction and renovation of the Grand Canal's water quality and environment have resulted in several benefits, including the following: Livelier banks: Improved water quality and a healthier living environment have led to more vibrant and lively canal banks. Residents and visitors alike can enjoy walks, cycling, and other outdoor activities along the canal, which has become a popular destination for recreation and tourism. More beautiful communities: The restoration and improvement of the Grand Canal have also contributed to the beautification of the surrounding communities. The canal banks are now adorned with flowers, trees, and other greenery, creating a more visually appealing and attractive environment. Better quality of life: Access to clean and safe water has had a significant impact on residents' quality of life. Improved water quality means residents can enjoy better health. Healthier living environment: The improved water quality and environmental conditions of the Grand Canal have also had a positive impact on the wider ecosystem. Fish and other aquatic life have returned to the canal, and the water quality has improved, reducing the risk of waterborne illnesses and pollution-related health problems. In conclusion, all Informats answered that the construction and renovation of the Grand Canal's water quality and environment have had far-reaching benefits, which made the banks of the Grand Canal more lively, their communities more beautiful, their lives replete with more quality, and their living environment more healthy.

## 5. Conclusion

“Green” has become a key component of the current and future urban development, and the same applies to a city with a rich cultural history. By combining the cultural heritage of the Grand Canal (Hangzhou section), this study identifies a successful example of the integration of the Grand Canal's cultural heritage conservation strategies into urban green development. Simultaneously, the surrounding citizens' living conditions and quality of life have improved as a result of the coordinated management of cultural heritage and green development in addition to enhancing their sense of wellbeing and satisfaction with the city.

### 5.1. Theoretical contributions

Green development and cultural heritage management are two important areas of research that have received significant attention in recent years. Theoretical contributions in these fields have played a crucial role in shaping our understanding of the complex relationship between economic development, cultural heritage preservation, and environmental sustainability.

Green development is an approach to economic development that emphasizes sustainability and environmental protection. The theoretical foundations of green development can be traced back to the concept of sustainable development, which was first introduced in the 1987 Brundtland Report. The aforementioned report defined sustainable development as “development that meets the needs of the present without compromising the ability of future generations to meet their own needs.” Since then, a large body of literature has emerged that explores the various dimensions of green development, including sustainable economics, green infrastructure, and sustainable urban planning.

Cultural heritage management is concerned with government attention, dynamic management, and policy support. Cultural heritage can include everything from historic buildings and archeological sites to traditional knowledge and cultural practices. The theoretical foundations of cultural heritage management can be traced back to the field of cultural anthropology, which has long been concerned with the study of cultural practices and traditions. Another important theoretical contribution is in the field of dynamic management. This concept emphasizes the importance of designing management strategies that enhance the resilience of cultural heritage resources, allowing them to withstand and recover from the impacts of environmental and social change.

### 5.2. Practical significance

The most striking aspect of this research is the innovative integration of traditional urban cultural heritage management, as an intrinsic factor in the green development of a city, into the contemporary concept of green development. By analyzing the conservation and upgrading of the Grand Canal (Hangzhou section), the macroscopic problem became concretized and the important value of the conservation and upgrading of historical and cultural heritage, in harmony with the green development of the urban, is argued.

Because of the Grand Canal's large spatial and temporal span, the diverse types of cultural heritage, the overlapping and interlocking heritage resources of different periods and forms, and the conservation requirements are currently more complex than those of general heritage. Some intangible cultural heritage inheritance vitality still needs to be improved. However, in some cases, a conflict exists between the conservation and utilization of natural resources involved in various types of culture and the green development of the Grand Canal.

Scholars have raised a number of concerns about the relationship between cultural heritage preservation and green development. Some of the key concerns include the following: Balancing economic development and heritage preservation: One of the main concerns is how to balance the need for economic development with the preservation of cultural heritage. Many scholars are worried that economic development can lead to the destruction of cultural heritage sites and practices. Another concern is that cultural heritage preservation may not receive adequate funding. Scholars are worried that, without sufficient resources, heritage sites may not be adequately protected and preserved. Scholars are concerned that heritage preservation efforts may not involve local communities enough. They are worried that, without the involvement and participation of local communities, preservation efforts may be less effective and may not reflect the cultural values and priorities of the people who live in the area.

Of course, from the information materials observed and gathered by the author, the local government of Hangzhou City and grassroots governments such as Gongshu District are comprehensively implementing the requirements of the 20th CPC National Congress, especially the new development concept proposed by General Secretary Xi Jinping, promoting the protection and utilization of the Grand Canal cultural heritage, and encouraging the harmonious development of humans and nature (6). Especially in conjunction with the Asian Games to be held in 2023, more conservation projects have been arranged to make cultural heritage protection and green development more coordinated and sustainable so that the local residents can get more benefits from them and have a stronger sense of access and happiness. Combining green development and urban cultural heritage can be a challenging task. It is important to preserve the existing urban cultural heritage to maintain a sense of history and place, and a balance needs to be struck between preserving the past and preparing for the future. It is important to recognize the unique history and character of each community or city and work to integrate green development in a way that enhances these qualities.

Specifically, the sample size used in the article's research, which implies that the results or conclusions are drawn from the data, may not be as reliable or generalizable as they could be with research done with a larger sample size. This limitation could impact the credibility and validity of the research presented in the article. For instance, a small sample size might not adequately represent the population being studied, leading to a potential bias and a lack of precision in the results. Additionally, it may limit the ability to draw meaningful and robust conclusions from the data, which could impact the usefulness of the article for readers.

## Data availability statement

The original contributions presented in the study are included in the article/supplementary material, further inquiries can be directed to the corresponding author.

## Author contributions

Conceptualization, formal analysis, methodology, resources, and writing: YX. The author has read and agreed to the published version of the manuscript.

## Footnotes

^1^Qinghai Province International Internet News Center. Study on green development by green culture. Qinghai News.

^2^Zhang Jianglin. Green development: a strategic concept of sustainable development.
